# Molecular docking analysis of an isoflavone derivative with the protein phosphatase 1 from Leishmania donovani 

**DOI:** 10.6026/97320630016942

**Published:** 2020-11-30

**Authors:** Rahila Qureshi, Malini Devi Alaparthi, Prathyusha Sai Eligati, Syed Rizwan Hasan Razvi, Komal Paresh Walvekar, Mohammad Afraa, Someswar Rao Sagurthi

**Affiliations:** 1 Drug Design & Molecular Medicine Laboratory, Department of Genetics & Biotechnology, Osmania University, Hyderabad, Telangana-500007, India

**Keywords:** Leishmania donovani, Resistance, Protein phosphatase 1, Isoflavonoids; Docking

## Abstract

Leishmaniasis is one of the most neglected diseases with high morbidity and mortality rate. Severe side effects with existing drug and lack of proper vaccine encouraged us to design alternative models to combat the disease. We showed that PP1 of Leishmania donovani mediates immunomodulation in host macrophages needed for parasite survival. Therefore, it is of interest to report the molecular docking analysis of 512 isoflavone derivatives with the phosphatase 1 protein from Leishmania donovani to highlight compound 362 (5-hydroxy-5-{9-[2-methoxy-2-(2-methylfuran-3-yl) ethyl]-1H, 3H, 4H, 10bH-pyrano[4,3-c]chromen-3-yl}pentanoic acid) having good binding features and acceptable ADMET properties for further consideration.

## Background

Leishmaniasis is a vector borne disease, caused by protozoan parasites of the genus Leishmania. This disease is recognized as a neglected tropical disease and mainly associated with poverty stricken areas with endemicity in more than 98 countries. Approximately 350 million people are estimated to be at risk of leishmaniasis, with an estimated 0.7 to 1 million new cases occurring annually [1]. The severity of the disease varies from disfiguring cutaneous form to the more fatal and life threatening visceral leishmaniasis. Leismania donavani causes visceral form and is transmitted by phlebotomine fly to the human host. The flagellated promastigotes differentiate into non-flagellated and pathogenic amastigotes after being phagocytized by macrophages. Non-availability of vaccine and limitations of chemotherapy as well as toxicity and emergence of resistance further aggravate the condition and press for the need of exploring important leishmanial proteins inhibitors which can also be exploited further for the development of antileishmanials [2]. To survive and differentiate in the hostile conditions inside the macrophage, a stress response through signal transduction gets initiated in the parasite [3]. Reversible phosphorylation through kinases and phosphatises is the critical event during the parasitic stress response and known to cause alterations in expression, interaction and activity profiles of various proteins [3]. The phosphatome and kinome analysis of various parasites from trypanosomatid family and species of Leishmania highlights the abundance of protein phosphatases maintaining phosphorylation-dephosphorylation equilibrium. Protein phosphatases dephosphorylate substrates and play an important role in posttranslational modifications [4], cellular differentiation [5] and drug resistance [6]. Almost 96–99% proteins in the eukaryotes proteome get phosphorylated on serine and threonine residues through ser/thr phosphatases [7]. PP1 group of ser/thr phosphatases is ubiquitously present in most of the cells and proven to be inhibited by known natural compounds such as okadaic acid, calyculin A, microcystin, tautomycin and cantharadin. Inhibition of PP1 in P. falciparum has resulted in hampered parasitic growth and its ablation through RNAi resulted in decreased DNA synthesis, confirming its role during replication of the parasite [8]. Additionally, calyculin A and okadaic acid mediated inhibition of PP1 advocated towards an important role played by PP1 during parasite’s attachment to their host cells in Trichomonas vaginalis [9], Toxoplasma gondii [10] and P. falciparum [11]. Similarly, inhibition of PP1 in T. brucei with the same compound resulted in defected segregation of kinetoplastids, stalled cytokinesis and interrupted organellar cycle [12]. Another detailed study evaluated inhibition of PP1 through tautomycetin and demonstrated the regulation of mitogen-activated protein kinase (MAPK) by interacting with Raf-1, hence act as a positive regulator of Raf-MEK-ERK pathway [13]. A small molecule like guanabenz and its derivative Sephin selectively inhibited the regulatory subunit of PP1 in vivo. Remarkably, Sephin1 does not inhibit the constitutively present form of PP1 regulatory subunit (PP1R15B) but specifically binds and inhibits the stress-induced form (PP1R15A) of the same and prevented diseases in mice related to protein misfolding [14]. Other mechanisms including blocking thePP1 regulatory subunit from accessing its physiological substrate or inducing disassembly of regulatory and catalytic subunits have also been suggested to achieve selective inhibition of PP1 holoenzyme [15]. Thus, ' owing to the indispensable role played by PP1, several inhibitors have been explored in higher eukaryotes including mice and human. However, in spite of being involved in many cellular processes, the fundamental mechanistic pertaining to the critical phosphatase inhibition is needed. Recently, we have characterized the PP1 of L. donovani (LdPP1) that shows protein-mediated immunomodulation in humanmacrophages. The three-dimensional structure of LdPP1, determined by homology modelling, displayed all thecharacteristic features corresponding to theknown PP1 structures. Binding of known inhibitor (okadaic acid) with LdPP1 has provided insights into the molecular mechanism of inhibition [16]. The isoflavones and their derivatives have been proven as leishmanicidal and anti-plasmodial activity [17]. Therefore, it is of interest to report the molecular docking analysis of 512 isoflavone derivatives with the phosphatase 1 protein from Leishmania donovani to highlight compound 362 (5-hydroxy-5-{9- [2-methoxy-2-(2-methylfuran-3-yl) ethyl]-1H, 3H, 4H, 10bH-pyrano [4,3-c]chromen‐3-yl}pentanoic acid) having acceptable ADMET properties for further consideration.

## Methodology:

### Compound dataset:

Isoflavonoids and their derivatives have been evidenced the multiple uses with low toxicity profile. Okadaic acid is a toxin produced by dinoflagellates, which inhibits human PP1. Combining the molecular binding profile of the two molecules we rationally designed a library of 512 molecules against LdPP1 by using MarvinSketch 5.6.0.2. The pharmacological efficiency of these molecules is further validated by okadaic acid, known inhibitor of protein phosphatses produced from sponges and shellfish.

### Protein homology modelling:

The structural characterization of LdPP1 has been reported earlier [16]. Briefly, homology modelling of the protein was performed using Modeller [18]. This generated five different models, of which best model with better stereo chemical properties was chosen through ModLoop [19]. Finally, the energy minimized structure required for docking was developed using the GROMACS program.

### Ligand and protein preparation:

All the designed molecules were cleaned and converted to three-dimensional form with explicit hydrogen bonds by using MarvinView application 5.6.0.2. Further, the molecules were saved in .pdb format for docking studies. Simultaneously, the protein was also prepared by assigning missing bonds and bond orders. Also, charges were assigned at necessary places and the final structure was saved in .pdb format.

### Molecular docking of Compounds:

Molecular docking provides information regarding how the ligand interacts with target protein at molecular level. In the present study, we used Molegro Virtual Docker 2010.4.0 to screen the compound dataset at docking site [20- 23]. A mathematical notation called Moldock score, which is based on Piecewise Linear Potential (PLP), gave the protein-ligand interaction. The total Moldock score represents the sum of total internal ligand energies and protein interaction energies along with soft penalties. Accordingly, the compound with highest Moldock score has highest binding energies with good protein-ligand interactions.

### ADMET profiling of compounds:

The druggable nature of the compounds cannot be predicted by ligand protein interactions alone. It should also possess good pharmacokinetic properties with low toxicity profile. Thus, complete ADMET profiling of the compounds was performed using ADMETSAR webserver.

## Results and Discussion:

**Figure 1:**
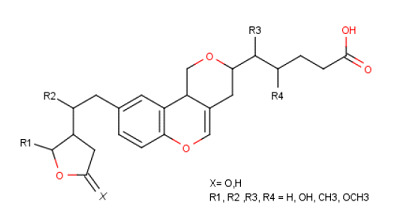
Basic scaffold of designed molecule with different substituents.

**Figure 2:**
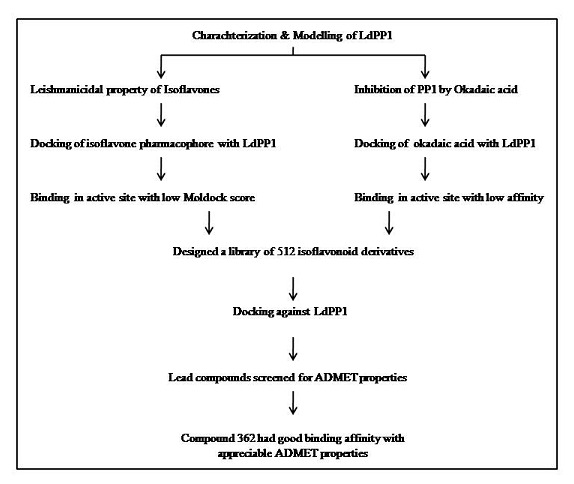
Flow chart outlining the steps in optimizing the lead molecule against LdPP1

**Figure 3:**
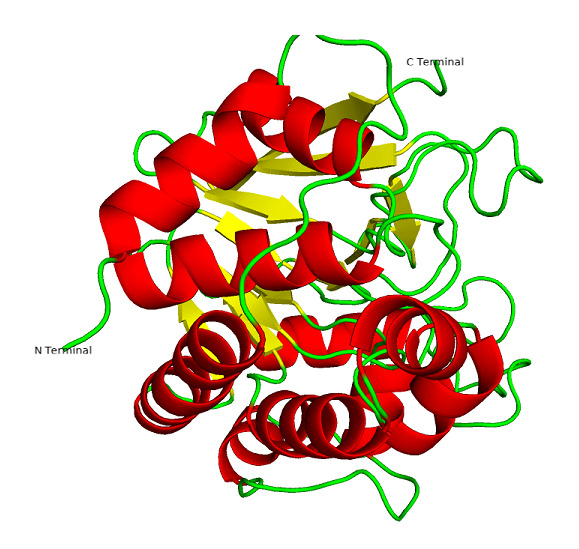
3D homology model structure of Ldpp1

**Figure 4:**
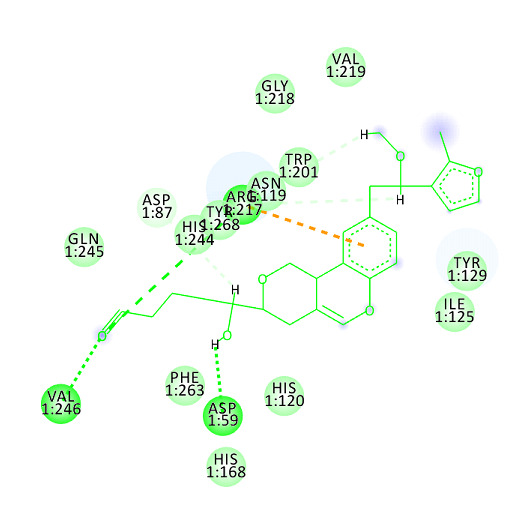
2D representation of ligand-receptor with LdPP1. Orange colour dotted line represents Pi-cation interaction; Light green colour thick dotted line represents conventional hydrogen bonds, whereas the light green colour thin dotted line represents carbon hydrogen bonds. The non-bonded free amino acids around shows van der Waals interactions.

Basic scaffold of the designed molecules along with different substituents at R1, R2, R3 and R4 was shown in the Figure 1. In the library (Table 1 - see PDF), few molecules have shown best affinity towards LdPP1, out of which top three molecules along with okadaic acid structures were given in the Table 2(see PDF). All the molecules have shown almost equal affinity towards LdPP1 as okadaic acid at the active site. Compound 362 has exhibited highest affinity with Moldock score of -148.38 and Rerank score -112.17 which is comparable to okadaic acid with comparable Moldock -141.45 and least rerank score as -85.86 (Table 2 - see PDF). The docking profile of compound 362, with energy descriptor values and their interactions in detail were given in Table 3(see PDF). In external ligand interactions, which include mainly protein-ligand interactions, the steric interactions provide major stability with value -131.05 based on piecewise linear potential. Along with, hydrogen bond interactions also play a key role in stabilizing the ligand protein duo. Optimal affinity of the molecules towards LdPP1 was contributed by different interactions namely van der Waals forces, conventional hydrogen bond, carbon hydrogen bond, Pi-cationic and Pi-alkyl bonds. Asp59, Asp87, Arg91, Asn119, His120, Ile125, Tyr129, His168, Trp201, Arg217, Gly218, Val219, His244, Gln245, Val246, Phe263, Tyr268 are the amino acids having various interactions with target protein. The lead molecule 362, demonstrates van der Waals interactions with Asp87, Arg91, His120, Ile125, Tyr129, His168, Gly218, Val219, Gln245, Phe263 conventional carbon hydrogen interactions with Asp59, Val246, Tyr268, carbon hydrogen bond with Asn119, Trp201, His244 and pi- cationic interaction with Arg217 (Figure 4). The lead molecules with highest affinity were assessed for their ADMET properties, and the result demonstrated well appreciable pharmacokinetic parameters i.e. absorption, distribution, metabolism and excretion as shown in Table 4(see PDF). All the compounds exhibited non-carcinogenic properties, which are comparable to okadaic acid. Figure 2,Figure 3 illustrates the steps involved in optimizing the lead molecule against LdPP1. The high affinity of isoflavone derivatives towards LdPP1 envisage a novel class of inhibitors in the treatment of leishmanial infections with low toxicity profile and druggable nature of compounds might support further research in this area to subside the leishmanial infection rate.

## Conclusion

We report binding structure feature data on compound 362 (5‐hydroxy‐5‐{9‐[2‐methoxy‐2‐(2‐methylfuran‐3‐yl) ethyl]‐1H, 3H, 4H, 10bH‐pyrano [4,3‐c] chromen‐3‐yl}pentanoic acid) with PP1 having acceptable ADMET properties for further consideration to combat the disease Leishmaniasis.
